# Dead Cas Systems: Types, Principles, and Applications

**DOI:** 10.3390/ijms20236041

**Published:** 2019-11-30

**Authors:** Sergey Brezgin, Anastasiya Kostyusheva, Dmitry Kostyushev, Vladimir Chulanov

**Affiliations:** 1 National Medical Research Center of Tuberculosis and Infectious Diseases, Ministry of Health, Moscow 127994, Russia; seegez@mail.ru (S.B.); kostyusheva_ap@mail.ru (A.K.); vladimir.chulanov@rcvh.ru (V.C.); 2 Institute of Immunology, Federal Medical Biological Agency, Moscow 115522, Russia; 3 Sechenov First Moscow State Medical University, Moscow 119146, Russia; 4 Central Research Institute of Epidemiology, Moscow 111123, Russia

**Keywords:** Cas9, dCas, transcription, epigenetics, chromatin, cancer, hereditary diseases, inflammatory diseases, infectious diseases, editing

## Abstract

The gene editing tool CRISPR-Cas has become the foundation for developing numerous molecular systems used in research and, increasingly, in medical practice. In particular, Cas proteins devoid of nucleolytic activity (dead Cas proteins; dCas) can be used to deliver functional cargo to programmed sites in the genome. In this review, we describe current CRISPR systems used for developing different dCas-based molecular approaches and summarize their most significant applications. We conclude with comments on the state-of-art in the CRISPR field and future directions.

## 1. Introduction

In recent years, precise genomic and epigenomic editing has transformed into a fast-growing area of research, with lucrative applications in medicine and biotechnology. Targeted modifications of genomes in various organisms, from bacteria to plants to mammals, can be applied to treating human diseases and to developing bacterial strains and genetically engineered organisms with desired properties. Discovery of site-specific CRISPR-Cas nucleases and adapting these bacterial tools for gene editing applications have revolutionized genetic engineering and molecular biology. Robust activity, easy design, and capacity to target virtually any DNA or RNA site has put CRISPR-Cas at the forefront of gene editing techniques, with yet-undiscovered potential applications of optimized CRISPR-Cas components and novel CRISPR-Cas systems. In simplest terms, CRISPR-Cas systems are based on nucleolytic activity of Cas9 protein guided by a chimeric RNA molecule (guide RNA; gRNA) to the desired site in the genome. An important property of CRISPR-Cas is the high specificity defined by gRNA sequences that recognize the nucleic acid target and the protospacer adjacent motif (PAM) sequence, adjacent to the target, and required for CRISPR-Cas activity.

**Figure 1:**
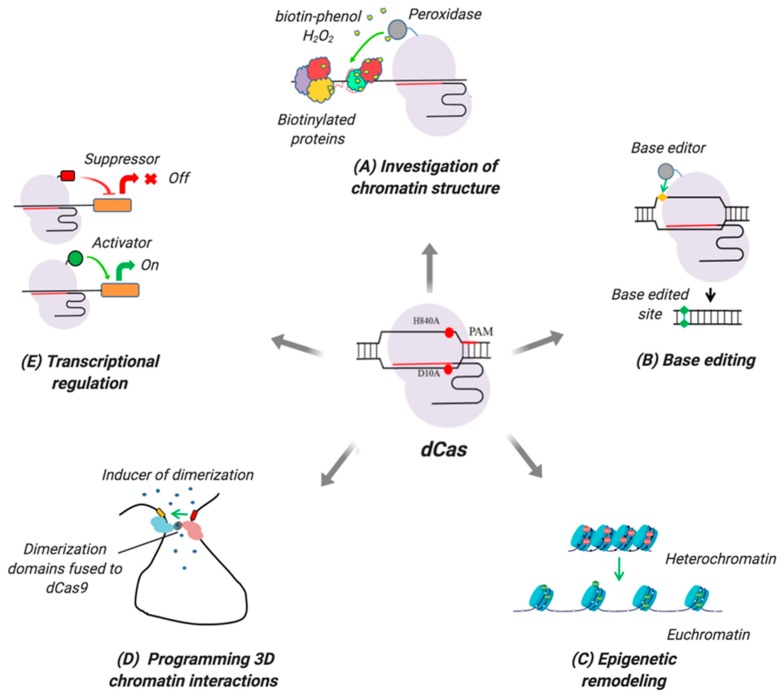
Types and applications of dCas-based molecular tools. (**A**) Investigation of chromatin structure. dCas proteins tethered with specific enzymes (e.g., peroxidase) enable inducible marking (biotinylation) of chromatin factors in the vicinity of the target site. These factors can be subsequently analyzed by proteomics to study chromatin organization. (**B**) Base editing. dCas proteins coupled with base editing enzymes (cytidine or adenine deaminases) can be used to modify RNA or DNA, correct genetic mutations, or knock-out genes. (**C**) Epigenetic remodeling. dCas-based epigenome modifiers can directly alter epigenetic state at a given locus, which is frequently used to annotate gene regulatory elements. Red and green spheres indicate heterochromatin and euchromatin marks, correspondingly. (**D**) Programming 3D chromatin interactions. Using two dCas proteins targeting defined genomic loci can program 3D chromatin interactions. A chemical inducer stimulates dimerization of dCas proteins fused with dimerization domains building long-range connections between genomic elements. (**E**) Transcriptional regulation. Control of gene expression by dCas proteins tethered to transcriptional suppressors (red) or activators (green). PAM—protospacer adjacent motif; H840A and D10A are point mutations inactivating catalytic residues RuvC and HNH, correspondingly. This picture was created in BioRender software.

Type II CRISPR-Cas systems are most commonly used, while CRISPR-Cas systems of other types (e.g., types V and VI) have also been leveraged for genomic and epigenomic editing. The Cas9 protein of the type II CRISPR-Cas system harbors two nucleolytic domains (RuvC and HNH) that cleave target DNA strands and generate double-stranded breaks (DSB) [1]. Introducing point mutations into each domain (D10A and H840A, correspondingly) blocks nucleolytic activity of Cas9 but does not impact its binding to its target [2]. This mutant protein, called dead Cas9 (dCas9), has significantly broadened the application of CRISPR-Cas9 technology. Chimeric dCas-X molecules, in which X is, in principle, any functionally active domain, can be used to deliver virtually any cargo (functionally active domains) to specific loci in the genome. Functionally active domains may include (a) epigenome remodeling factors for activating or suppressing gene expression; (b) domains for investigating chromatin structure; (c) domains for directly remodeling three-dimensional (3D) chromatin structure; and (d) base editing enzymes, among others (Figure 1).

In this review, we will discuss modifications of dCas proteins and the corresponding molecular techniques, principles of gRNA design, and applications of dCas-based technologies, as well as future developments.

## 2. Epigenomic Remodeling Using dCas-X

Activity of genes is mainly determined by chromatin architecture in regulatory regions (promoters and enhancers). Chromatin exists in two major forms: Transcriptionally inactive heterochromatin and active euchromatin. Epigenetically modifying DNA or histones alters chromatin state. Controlling the processes of epigenomic remodeling determines gene transcription. dCas proteins can be designed to directly modify DNA epigenetics by methylation or demethylation, as well as to change epigenetic marks on histones by acetylation/deacetylation, methylation/demethylation, or recruitment of transcription factors. Different types of dCas-X proteins and their potential applications are reviewed below. Manipulating epigenetics is needed to studying gene function and regulatory genetic elements, as well as for developing potential therapeutics that, for example, suppress viral genome activity, modulate immune responses, suppress oncogenes, or program stem cells.

### 2.1. Modifying the Methylation State of DNA

Methylation of DNA in regulatory regions of genes suppresses gene transcription [3]. DNA methyltransferase DNMT3A and its cofactor DNMT3L methylate DNA [4], whereas TET1 protein initiates removal of methyl residues from DNA [5].

dCas9-X technology has, for the first time, provided an opportunity to perform precise epigenetic DNA modifications. One of the first CRISPR-based tools to directly methylate DNA were chimeric proteins dCas9-DNMT3A and dCas9 linked to a catalytic domain of DNMT3A [6,7,8]. Efficacy of dCas9-DNMT3A was further enhanced by binding DNMT3A to a DNMT3L cofactor via a short peptide linker (dCas9-DNMT3A-3L). Adding DNMT3L to the system resulted in 4–5-fold increase in methylation efficacy and the size of the methylation editing window [9].

Another modification of dCas-based technology designed to increase methylation efficiency is coupling dCas-X with the SunTag system, which recruits multiple effector molecules (e.g., DNMT3A) to the desired site. The principle of dCas-SunTag is attachment of GCN4 peptide repeats to dCas9 and simultaneous intracellular production of effector molecules fused with single-chain variable fragments (scFv) that are prone to interacting with GCN4-dCas9. Several (up to 10 units) DNMT3A-scFv molecules bind to dCas9 via GCN4 repeats [10].

dCas9-SunTag-DNMT3A is highly specific, and does not seem to affect the overall methylation state of the genome [10]. It is appropriate for precisely methylating small portions of the genome to, for example, prevent interactions between genomic DNA and small interacting proteins. Systems linked to SunTag or DNMT3A-3L systems can be used to methylate large swaths of the genome and substantially suppress gene transcription. Whilst much more effective, the latter systems have multiple components and are very large, and thus can be difficult to accommodate into commonly used adeno-associated viral (AAV) vectors and other viral delivery tools.

In contrast, many research and potential therapeutic applications frequently require demethylation of genomic DNA, particularly to treat malignancies by activating tumor suppressors, to treat genetic diseases, or to generate or differentiate stem cells. Chimeric dCas9 proteins fused with a domain of TET1 DNA-demethylase (dCas9-TET1 or dCas9-SunTag-TET1) have been shown to effectively demethylate up to 90% of target DNA regions [8,11,12]. A different implementation of this technology includes three components: (1) dCas9 protein without additional domains; (2) gRNA with two special MS2-hairpins (aptamers); and (3) effector proteins linked to MCP proteins that interact with MS2. In this system, dCas9 protein in complex with a modified gRNA is first recruited to the target genomic region. TET1-MCP proteins then recognize and bind to MS2 hairpins within gRNA, enabling site-specific demethylation. Two molecules of MCP can interact with 1 MS2 hairpin, and a single gRNA harbors 2 MS2 hairpins, resulting in up to four units of TET1 attracted with a single modified gRNA [13].

### 2.2. Principles of gRNA Design for Genome Methylation or Demethylation

gRNA design is one of the most important considerations affecting CRISPR-Cas function. Several factors determine effective on-target mutagenesis induced by nucleolytic CRISPR-Cas systems, including availability of the PAM sequence, gRNA nucleotide (nt) composition, and the similarity between the gRNA and its DNA target. Additionally, designing gRNAs for methylation/demethylation applications requires several other parameters to consider:

- Initial methylation of target DNA. Effective suppression of gene transcription by dCas9-DNMTs can be achieved if gRNAs target initially unmethylated or weakly methylated regions [8]. In contrast, dCas9-based systems of DNA demethylation are effective only if gRNAs target heavily methylated DNA regions. DNA methylation levels in different cell lines and tissues can be assessed using several databases, including ENCODE [14] and MethBase [15].
- Methylation sites. Using dCas9-DNMT3A results in methylation of two regions. The first one lies within 27 nt in the 3ʹ-direction from the PAM sequence, and the second is within 27 nt from 5ʹ-end of the gRNA. The site of dCas9 binding (approximately 30 nt) is not methylated [6,9]. Methylation at the two sites occurs due to a flexible peptide linker between the dCas9 protein and the DNMT3A enzyme/catalytic subunit, providing mobility to the methyltransferase enzyme [6]. Peaks of DNA methylation vary upon introducing additional factors to the system, such as binding DNMT3A to DNMT3L [9].
- Methylation window. Methylation of extensive DNA regions is mandatory for stable suppression of gene function. dCas9-DNMT3A methylates regions 25–35 nt in length [6], and thus can only be used for pinpoint methylation [8]. Methylating extensive DNA regions is possible when using multiple gRNAs annealing to proximal DNA regions with the dCas9-DNMT3A system, [6] or a single gRNA combined with dCas9-DNMT3A-3L systems (which can methylate up to 1000 nt) [9] or dCas9-SunTag-DNMT3A (which methylates up to 4500 nt) [10]. DNA demethylation can be induced by dCas9-SunTag-TET1 within 200 nt of dCas9 binding (±100 nt from the dCas9 binding site) [12].

### 2.3. Regulating DNA Methylation State by dCas-Based Tools: Practical Applications

dCas-DNMTs tools provide an opportunity to analyze gene function and the role of methylation in physiologic conditions and in disease. For instance, dCas9-based methylation tools helped to identify the association between promoter methylation and dysfunction of *Desmoplakin* gene expression in the pathogenesis of idiopathic lung fibrosis [16]. Moreover, methylation of enhancer regions regulating phospholipidphosphatases was shown to be associated with calcification of the aortic valve, a pathological condition leading to myocardial infarction [17]. dCas9-TET1 helped to identify the key role of *TNFα* promoter hypermethylation in the development of nephropathies in diabetes [18]. The role of multiple genes in the processes of cell transformation was investigated using the dCas9-DNMT3A system. Directly hypermethylating DNA regulatory regions of tumor suppressors, including *CDKN2A, RASSF1, HIC1, PTEN*, and *SMARCA2*, improved understanding of gene function in oncogenesis [19,20]. The results of CRISPR-mediated methylation of tumor-related genes are summarized in the MICMIC resource [21]. Generating dCas9-DNMT3A tools aided analysis of gene transcription regulation by a small CTCF factor [8]. dCas9-based methylation was also used to impair CTCF-linked interaction of the *MYC* proto-oncogene with its super-enhancer and thus was proposed as an effective approach to treat many oncological diseases [22]. Additionally, *BRCA* [11] and *SARI* [23] promoters were demethylated to normalize physiological expression of tumor suppressors.

dCas9-methylation and demethylation tools can be leveraged to develop new therapeutic approaches performed at the level of epigenetics. Demethylating *FMR1* by dCas9-TET1, for example, was shown to correct the clinical manifestations of fragile X syndrome [24]. Moreover, hypermethylating the *SNCA* gene promoter led to decreased cell death in an in vitro model of Parkinson’s disease and thus can be potentially considered a new therapeutic approach [25].

The growing area of stem cell research and its application in regenerative medicine requires new, more advanced techniques to obtain pluripotent stem cells, maintain pluripotency, and differentiate stem cells into particular lineages. Recently, demethylation of *Sox1* gene by dCas9-TET1 resulted in efficient reprogramming of neural stem progenitor cells [26].

## 3. Rewriting Histone Epigenetic Marks

In addition to DNA methylation, histones are another factor involved in transcriptional regulation. Heterochromatin formation includes several steps: (1) Deacetylation of H3K9 and H3K27 histone residues; (2) methylation of H3K9 and H3K27 (H3K9Me3 and H3K27Me3); and (3) methylation of DNA regions wrapped by histones [27]. Histone deacetylation is catalyzed by histone deacetylases, methylation of H3K9 is executed by proteins SUV39H1 and G9A [28,29], and H3K27 is methylated by EZH2 [30]. Histone deacetylation and methylation suppress gene transcription [31,32,33,34].

On the other hand, transcriptionally active chromatin (euchromatin) is characterized by methylated H3K4 and H3K79 histone residues and acetylated H3K9 and H3K27 residues [35,36]. Factors MLL and PRDM methylate H3K4, while histone methyltransferase DOT1L attaches methyl groups to H3K79 residues [37]. Histone demethylation is mediated by LSD1 [38]. Acetylation of H3K9 and H3K27 is carried out by CBP and p300 histone acetyltransferases [39].

Targeted modifications of epigenetics in regulatory regions or in-site recruitment of transcriptional factors is the basis of CRISPR interference (CRISPRi) and CRISPR activation (CRISPRa) approaches. CRISPRi/a approaches rely on dCas proteins linked to functional activating or repressing domains.

### 3.1. CRISPRi

Current techniques enabling manipulation of gene activity include siRNA/shRNA approaches, which lead to degradation of transcribed mRNAs, and cDNA overexpression approaches. Several important drawbacks limit the application of these techniques. For example, siRNA/shRNA approaches frequently show significant off-target activity [40], and exogenous vectors have limited packaging capacity and can produce only a selected isoform of the gene of interest [41]. The latter may result in both qualitative and quantitative differences between the effects of a single gene isoform and many isoforms expressed from the genome.

Typically, CRISPRi is based on chimeric dCas-X proteins, where X is a repressive Krueppel-associated box (KRAB) domain [42] or enhancer of Zeste homolog 2 (EZH2) [34]. KRAB is a transcriptional repressor of eukaryotic genes; dCas9 molecules carrying KRAB target regulatory regions of genes (promoters or enhancers) [43] and attract histone deacetylases and methyltransferases that add epigenetic marks of inactive heterochromatin H3K9 and H3K27 (or H3K27 for dCas9-EZH2) [34], ultimately blocking mRNA synthesis [42,44].

Both dCas9-KRAB and dCas9-EZH2 affect genes transiently. Sustained suppression of transcription is possible if two systems (dCas9-KRAB/dCas9-EZH2) are combined with dCas DNA methylation systems (dCas9-DNMT3A-3L [34,45,46] or dCas9-SunTag-DNMT3A [10]). Alternatively, a repressive dCas9-KRAB-MeCP2 system can be used, as MeCP2 attracts DNMTs and histone deacetylases independently of KRAB. This combined system has 4-fold higher transcriptional repressor activity than KRAB system alone [47].

Lysine-specific demethylase LSD1 can be used for transcriptional repression as well. Gene silencing by dCas9-LSD1 is based on the demethylating active H3K4Me3 residues followed by H3K27 deacetylation [48]. LSD1-mediated regulation is enhancer-specific [48]. dCas tools fused with LSD1 are used to annotate unknown distal regulatory elements, as LSD1 activity is limited to enhancers. EZH2 and KRAB domains are comparable in repressive efficiency, but KRAB is more widely used and historically is one of the first transcriptional repressors adopted for precise epigenomic modifications.

### 3.2. CRISPRa

Similarly, CRISPRa approaches take advantage of dCas proteins to recruit activation domains to regulatory genomic elements and induce target gene transcription. First mammalian CRISPR activators were based on chimeric dCas9 proteins fused with p300 [49], p65 or p65 with heatshock factor 1 (HSF1) [50,51], or VP(16)_n_ [50,52,53]. These CRISPRa systems function by acetylating histones in target regions (catalytic subunit of p300 histone acetyltransferase) or directly activate genes by recruiting transcription factors (endogenous transcription factors p65 (a subunit of NFkB) or p65-HSF1).

Another scenario is to utilize dCas to deliver herpesvirus factor VP(16)_n_ (where *n* is the number of monomers) to regulatory elements, thereby recruiting preinitiation complex factors and activating target gene transcription. Gene activation by a VP16 monomer is very ineffective, so CRISPRa is usually coupled with multimers of VP16 (VP48, VP64, VP160, VP192) [54].

Robust induction of gene transcription by these systems requires multiplex gRNA targeting an extended genomic region. The VPR system relies on dCas9 protein linked to the protein complex VP64-p65-Rta, where Rta is an Epstein–Barr virus transcription factor. A three-component complex, VP64-p65-Rta has considerably higher efficacy than previous CRISPRa systems, potently activating target gene transcription [55,56].

**Figure 2:**
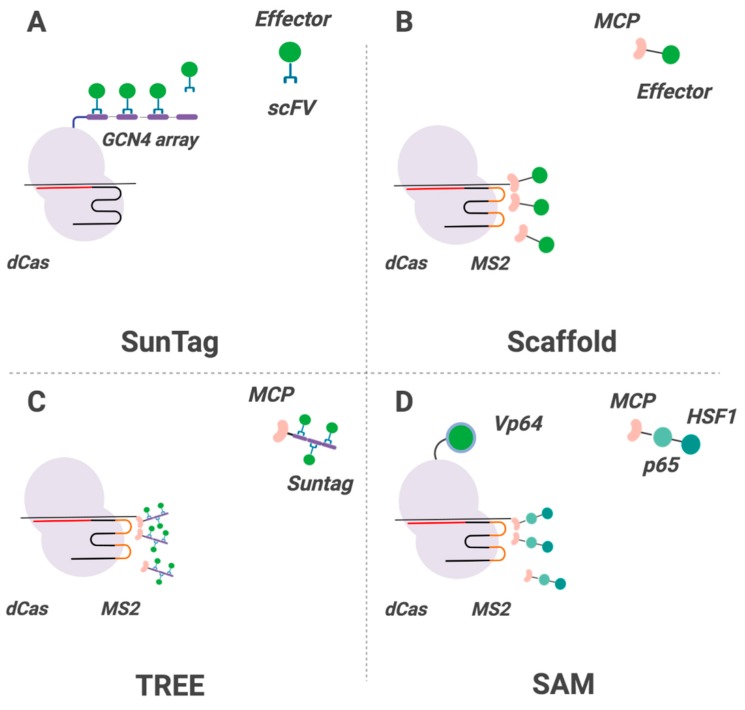
Modification of CRISPR components for improved epigenetic regulation. (**A**) SunTag technique. dCas is fused with GCN4 peptide array, which attracts any effector molecule containing single-chain variable fragments (scFV). Multiple GCN4-scFV interactions ensure efficient recruitment of many effector molecules to the dCas-programmed genomic site. (**B**) Scaffold technique. In Scaffold, effector molecules are recruited to the target site via the interaction of MCP aptamer-specific protein with a short synthetic gRNA containing MS2 aptamer. gRNA protrudes out of the Cas-gRNA complex, so that chimeric gRNA-MS2 transcripts efficiently recruit effectors carrying MCP molecules. (**C**) TREE combines SunTag and Scaffold techniques, providing additional recruitment of effector molecules. (**D**) SAM is based on a two-component transcriptional effector (p65-HSF1) recruited to the target via MS2-MCP interaction. Additionally, dCas protein is tethered to a transcriptional regulator (VP64) to increase potency of the effect. This picture was created in BioRender software.

CRISPRa systems can be coupled with affinity-binding technology that enables simultaneous recruitment of multiple domains to the target site. In this setting, different CRISPRa modifications can be introduced. These can modify Cas proteins (e.g., with SunTag technology), targeting gRNAs (e.g., Scaffold, Casilio), or both (SAM, TREE). These modified CRISPRa systems demonstrate high efficacy even with a single gRNA [57]. SunTag was described above; briefly, Cas9 is fused to a GCN4 peptide array that attracts scFv-linked pro-activation domains (VP64, p65-HSF1, p300, and others) [58,59] (Figure 2). Scaffold operates on a different principle; modified gRNAs carry aptamer sequences (MS2, PP7, or com) and attract aptamer-specific proteins (MCP, PCP, Com) fused to transcriptional activators [60] (Figure 2). The Casilio system is an upgrade of Scaffold and introduces the shorter *Casilio* aptamers into gRNA, improving gRNA stability and potency [61]. SAM combines dCas9 proteins linked to transcriptional activators and Scaffold technology with modified gRNAs, thus enabling transcriptional regulation both by transcriptional domains linked to dCas9 and domains recruited by gRNA aptamers [51,60] (Figure 2). The TREE system combines SunTag and Scaffold, enabling recruitment of up to 32 molecules of VP64 or p65-HSF1 [62].

Another implementation of CRISPRa is drawing an active cytomegalovirus (CMV) promoter to the target to activate regulatory regions of genes. This modification of CRISPRa is based on a hybrid gRNA molecule linked to a double-stranded DNA of the CMV promoter [63].

CRISPRa technologies are summarized in Table 1.

### 3.3. Principles of gRNA design for CRISPRa and CRISPRi

An important parameter defining the efficacy of CRISPRa/i systems is the epigenetic state of the target region and baseline gene expression levels. The following criteria should be taken into account when designing CRISPRa/i systems.

#### 3.3.1. CRISPRi

- Target region. CRISPRi approaches should primarily target proximal promoters or enhancers. gRNAs targeting promoters should be designed to anneal at –50 to +300 nt from transcription start site. Highest efficacy has been demonstrated for gRNAs targeting +50 to +100 nt [64]. Transcription start sites can be visualized using FANTOM5 [65] or GeneHancer databases [43].
- Epigenetic state. The most effective binding of dCas proteins occurs in areas of open chromatin determined by peaks of DNase I sensitivity [66]. Moreover, effective interference is observed when using gRNAs targeting sites enriched with marks of active chromatin (H3K27Ac, H3K9Ac, H3K4Me3, H3K4Me2, H3K79Me2) [67]. Epigenetic marks and sites of DNase I hypersensitivity can be monitored using ENCODE database [14].

#### 3.3.2. CRISPRa

- Target region. CRISPRa should target proximal promoters, or, for some systems (e.g., dCas9-p300), distal enhancers. CRISPRa gRNAs to promoters should be designed to interact within −400 to −50 nt from the transcription start site [49].
- Epigenetic state. The most effective activation of genes occurs when CRISPRa are recruited to the sites of DNase I hypersensitivity [66].

Design of gRNAs for many CRISPR application is made convenient by various online resources (reviewed in [68]).

### 3.4. Applications of CRISPRa/i

CRISPRa and CRISPRi are increasingly used in biological studies and in development of potential medications. These approaches have been extraordinarily effective for treating metabolic disorders and diseases associated with gene malfunction, including diabetes mellitus, Duchenne muscular dystrophy (DMD), and haploinsufficiency-related disorders, as well as for generating and differentiating stem cells.

As KRAB and p300 can repress and activate enhancers, these domains are used for dCas9-based screening of proposed distal regulatory elements and enhancers bound by transcriptional factors [69,70].

Shortly after they were described, CRISPRa approaches were used to activate endogenous antiviral factors *APOBEC3B* and *APOBEC3G* to combat human immunodeficiency virus (HIV) infection [71]. Another possible CRISPRa-based strategy to combat HIV infection is a “shock-and-kill” approach, which works by reactivating latent HIV using CRISPRa followed by death of infected cells by direct cytotoxic effects of the viral proteins or by immune clearance [72,73,74,75]. Most recently, a CRISPRa approach based on many CRISPRa systems was employed to activate *APOBECs* and destroy hepatitis B virus (HBV) genomes [76]; this method can be used in combination with Cas9 nucleases to treat HBV [77,78]. CRISPRs can also be used to identify new antiviral factors in cell models [79].

CRISPRa can help treat haploinsufficiency-induced diseases by overexpressing an intact copy of the insufficient gene. This method has been utilized in vivo to treat obesity [80] and Dravet syndrome [81].

CRISPR-mediated transcriptional regulation has great potential in oncology. CRISPRi approaches have been used to repress proto-oncogenes like *Granulin* [82], while activating tumor suppressors *PTEN, DKK3,* or *CHEK2* greatly suppresses proliferation of cancer cells [83,84,85]. A recently described MAEGI approach overexpresses tumor antigens by CRISPRa to increase their presentation to the immune system resulting in efficient destruction of tumor cells by cytotoxic CD8+ lymphocytes [86].

The most important examples of CRISPRa/i applications are summarized in Table 2.

## 4. Analyzing Factors Involved in Chromatin Remodeling

Chromatin structure in regulatory regions determines transcriptional activity of genes. Chromatin state is regulated by complex interactions between DNA, transcription factors, and associated RNAs. Identifying factors implicated in these interactions is important for understanding fundamental aspects of transcriptional regulation.

Typically, factors interacting with chromatin at a given site are studied using the chromatin immunoprecipitation (ChIP) technique and its modifications. However, ChIP has several drawbacks. It can be used to capture pre-defined transcription factors using specific antibodies but identifying new proteins and RNAs is difficult. Moreover, antibodies to pre-defined factors are limited and very expensive. Therefore, new techniques are needed to identify interacting partners of chromatin-remodeling complexes.

### 4.1. dCas Technology for Analyzing Chromatin-Remodeling Factors

In recent years, three dCas-based methods (CAPTURE, CasID, and CASPEX) were devised to identify previously unknown factors interacting with chromatin by means of high-affinity extraction and proteomics.

In the CAPTURE method, a dCas9 protein contains a site for a biotintransferase enzyme that is added to cells to transfer biotin markers onto dCas9. Biotinylated dCas9 proteins complexed with adjacent proteins at the site of interest are then isolated using avidin-streptavidin interactions [117]. CasID technology relies on biotinylation of all proteins in the vicinity of a chimeric dCas9 protein linked to biotintransferase [118]. In both CAPTURE and in CasID methods, biotinylated proteins are isolated using avidin-streptavidin and assayed qualitatively and quantitatively by proteomics (high-performance liquid chromatography-mass spectrometry and Western blotting), whereas RNA and DNA interacting with the site of interest can be identified by next-generation sequencing [117,118]

CAPTURE and CasID require long incubation steps (several hours) to generate biotinylated targets, and thus cannot be used to analyze dynamic processes of chromatin remodeling, epigenetic modifications in response to exogenous stimuli, cell differentiation, and cell cycling, among others. This limitation can be overcome by using the CASPEX techniques (C-BERST and GLoPro protocols), in which a dCas9 protein linked to APEX2 peroxidase is added to cells with a reaction mixture composed of hydrogen peroxide and biotin-phenol. APEX2 induces oxidation of biotin-phenol, generating short-lived, highly reactive free radicals that biotinylate all factors in the direct vicinity of dCas9-APEX2 binding (±400 nt), which can be further analyzed by proteomics or sequencing [119,120]. CASPEX can be used to analyze transient chromatin interactions but should be tightly controlled by regulating intracellular concentrations of dCas9-APEX2 to avoid labeling off-target proteins. dCas9-APEX2 levels can be regulated by binding dCas9 to degrading domains FKBP and L106P and by using inducible promoters [120].

### 4.2. Principles of gRNA Design for CAPTURE, CasID, and CASPEX Methods

- Target site. gRNAs should anneal at the most proximal area of the target region, but should not lie at sites bound by transcription factors to avoid impeding interactions between regulatory DNA elements and proteins [117].
- Off-target interactions. For better consistency, proteome analysis of chromatin architecture should be performed with validated negative controls (cells without dCas9, and cells with dCas9 but without gRNA) and should consider endogenous and non-specific biotinylation [117,118,119,120]. Generating several gRNAs for each site and further comparing the data are strongly recommended to discern factors stably bound to the target region and those with rare and transient interactions [117,118].

## 5. dCas Systems for Shaping Three-dimensional Chromatin Architecture

Distal regulatory elements are located many thousands of nucleotides from gene promoters but strongly impact gene transcription when drawn close together. Dysregulation of these processes results in aberrant gene expression and is frequently observed in human diseases, including cancer [121]. Identifying distal regulatory elements and elucidating their function is necessary to understand many physiological and pathological processes and is critical in drug design.

The CLOuD9 method involves interactions between two orthologous dCas9 proteins fused to dimerizing domains PYL1 and ABI1. One dCas9 protein interacts with a distal region, while the other targets the promoter of the gene of interest. Adding the inducer (abscisic acid) promotes dimerization of dCas9 proteins carrying dimerization domains and interaction of the bound chromosomal regions. CLOuD9 can be devised to directly manipulate the 3D architecture of chromatin, analyze distal regulatory regions, and install new intra- and inter-chromosomal links [122].

Optimal sites of gRNA targeting for CLOuD9 can be selected in genomic browsers with the described considerations for distal regulatory regions annotated in FANTOM5 [65] and GeneHancer [43] databases.

## 6. Editing Nucleic Acids

Many hereditary diseases, cancers, and mutations resulting in drug resistance are associated with single-nucleotide polymorphisms (SNPs) [123]. Editing SNPs by classic nucleolytic Cas proteins and homologous templates depends on complex DNA repair pathways and appears to be inefficient [124]. Simply correcting SNPs has become possible with dCas systems fused to nucleic acid editing factors.

### 6.1. DNA Editing Using dCas Tools

Base-editing systems leverage dCas proteins fused to cytidine or adenosine deaminases. Cytidine deaminases convert cytosine to uracil (C→U) with the resulting U•G mismatches being resolved by DNA repair machinery to form thymine (C→T) on the target strand and guanine (G→A) on the complementary strand [125,126], while adenosine deaminases deaminate A yielding inosine (I). I preferentially base-pairs with cytidine in the context of a polymerase active site; in the third position of tRNAs, anticodon I base-pairs with either A, U, or C during mRNA translation [127]. Recently, evolved adenine base editors able to effectively convert A•T base pairs into G•C base pairs in DNA have been described [128]. rAPOBEC1 [128], APOBEC3A [129], AID, and its homologues [130,131,132,133] are among the most widely used cytidine deaminases. Adenine deamination is done by TadA adenosine deaminase [134].

Efficacy of cytosine base editors may be fairly low due to repair of edited nucleotides by endogenous DNA repair systems like the UNG factor. Improved efficacy of base editing was shown for nickase nCas9 (nCas9) proteins, which have a single mutated nucleolytic domain and one domain with preserved cleavage activity, linked to base-editing factors and co-expressed with UGI, an UNG inhibitor [128]. Blocking UNG by UGI transiently impairs DNA repair so that deaminated nucleotides are not corrected. The nCas9 protein fused to base editing factors deaminates nucleotides at the target DNA strand and at the same time makes single-stranded DNA cuts (nicks) on the complementary strand [128]. Next, a 2–12 nt site adjacent to the nick is incised from the DNA, removing the template for error-free repair of the deaminated nucleotide at the target DNA strand. Combining nCas9 with UGI provides a robust base-editing platform for efficient and site-specific introduction of single-nucleotide mutations [128,133].

Correcting virtually any mutation in any DNA region has become possible by using genetically engineered dCas proteins with optimized (shortened) PAM motifs. Relieving PAM restrictions broadens the range of potential sites edited by base editing factors and provides an opportunity to treat numerous genetic disorders [135,136].

The major consideration when using dCas base editors is potential off-target editing of RNA, as was unexpectedly described for rAPOBEC1, TadA, and APOBEC3A [137,138,139]. Direct mutagenesis of deaminases opens new avenues for improving their activity and making safer analogs of base-editing factors [137,138,139,140].

Among cytidine deaminases, rAPOBEC1 possesses the highest deamination activity [128]. However, cytosine editing in a GC-rich context by rAPOBEC1 is very limited [128]. Optimized rAPOBEC1 base editors evoAPOBEC1-BE4max and evoFERNY overcome this limitation [141]. Additionally, rAPOBEC1 and AID-based editors perform poorly on heavily methylated DNA. An alternative is APOBEC3A enzyme, which is less sensitive to methylated DNA bases and, consequently, can be utilized to target methylated sites [129].

### 6.2. Editing RNA with dCas Tools

Effector proteins of CRISPR-Cas type VI systems can directly interact with target RNAs independently of PAM, thus allowing deaminases binding to these proteins to edit RNA molecules.

The REPAIR RNA-editing system is based on a dCas13b protein linked to a mutant form of ADAR2, an enzyme that catalyzes adenine deamination in dsRNA (A→I conversion) [142]. Using the method of directed evolution, a new variant of ADAR2 protein with enhanced properties was created to become the basis of the RESCUE system. In addition to adenine deamination, this optimized ADAR2 modifies cytosine nucleotides (C→U conversion) as well [143]. The REPAIR system is rather specific and does not exhibit significant off-target binding or RNA editing [142], with a relatively low number of off-target sites [143].

Compared to DNA editing, RNA editing has several important advantages, including a wider range of potential sites due to PAM-independent functioning of CRISPR-Cas type VI proteins and direct RNA editing by deaminases without the assistance of endogenous repair systems [142]. DNA editing has PAM restrictions so that canonical SpCas9-derived base editors do not ensure targeting of even a quarter of all known pathogenic SNPs. Tethering base editors with orthologous Cas proteins or engineered Cas proteins with modified PAM compatibilities may solve this problem [133,135]. Another advantage of editing RNA instead of DNA is that DNA editing yields indels. Fusing cytosine editors to the bacteriophage Mu-derived GAM or inhibiting UNG reduces the rates of indel formation [133]. Proximal (within 200 bp of the target site), bystander editing executed by deaminase domains, single-stranded DNA and RNA editing by random encountering with deaminases and distal off-target edits related to off-target binding of Cas proteins is an important issue and an area of active investigation. Developing mutated deaminases with context-dependent activity and modified editing windows coupled with more specific Cas proteins has the potential to reduce off-target mutations. In particular, REPAIRv2 system had a 900-fold reduction in off-target editing, but at the expense of on-target editing efficiency (almost 2-fold decline) [142]. Improvements in CRISPR-Cas components and base editing enzymes will pave the way for developing safer and more accurate tools, but it is unlikely that the off-target mutagenesis can be avoided completely. A detailed review of base editors was provided in a brilliant paper by H. Rees and D. Liu [127].

### 6.3. Applications of dCas Base Editors

Site-specific base editors can be used to correct mutations associated with a disease phenotype or to introduce mutations to block or modify gene function.

Precise base editing using CRISPR-STOP and iSTOP has become a novel tool to knock out genes by editing four potential triplets of nucleotides to generate stop codons. Introducing stop codons into the early exons of genes leads to synthesis of short, non-functional mutant proteins [144,145]. Unprecedented efficacy was observed when using iSTOP technology coupled with the SunTag system [146]. CRISPR-STOP and iSTOP do not rely on nucleolytic cleavage of DNA and DSB generation, and thus can be considered a safer alternative for knocking out genes compared to canonic CRISPR-Cas cleavage tools. The database for iSTOP includes 3.4 million gRNAs targeting 97%–99% of genes in eight eukaryotic species [145]. These methods have been immensely effective in massive loss-of-function screens, in developing disease models, and as perspective therapeutic tools.

dCas base editors were used to create in vitro and in vivo models of different diseases associated with SNPs. For instance, base editors were used to create cell lines resistant to chemotherapeutic drugs [130,132,147] and models of hereditary diseases (including DMD, X-linked dilated cardiomyopathy, and albinism) [134,148,149,150,151,152] and chronic diseases [153], as well as to analyze SNPs associated with malignant cell transformation and cancer [147].

Base editors proved useful for creating new therapeutic approaches. dCas-guided base editors corrected SNPs linked to hereditary diseases, such as thalassemia [154,155], Marfan syndrome [156], and phenylketonuria [157]. Introducing inactivating mutations into the *Pcsk9* gene was proposed as a therapeutic approach to treat atherosclerosis [158,159].

Conversely, an approach called CRISPR-Pass can be used to correct nonsense mutations by adenosine editors. Editing codons with nonsense mutations using CRISPR-Pass helps to recover production of active proteins. CRISPR-Pass was predicted to correct up to 95% mutations described in ClinVar database [160]. dCas-base editors were patented as antiviral tools capable of introducing mutations into viral genomes to block replication and protein synthesis of viruses including HIV, HBV, human papilloma virus, and Epstein–Barr virus.

CRISPR-SKIP method was created based on dCas base editors to introduce point mutations into splice acceptor sites. Generated mutations result in exon skipping and translation of new protein isoforms with altered properties [161]. This protocol can be used to investigate diseases linked to exon skipping or as therapeutic approaches for diseases like DMD. Most important applications and properties of dCas9-base editors are listed in Table 3 and Table 4, correspondingly.

### 6.4. Principles of gRNAs Design for Base Editing Applications

dCas base editing systems are characterized by a unique editing window. The majority of systems have a 5-nt window, which may vary if systems undergo modifications or are genetically optimized. Easy-to-use software, including BE-Designer (http://www.rgenome.net/be-designer/) and BE-Analyser (http://www.rgenome.net/be-analyzer), has been created to design gRNAs for base-editing applications [162]. For convenient design of gRNAs for correcting pathogenic T-to-C single nucleotide variations, BEable-GPS database (http://www.picb.ac.cn/rnomics/BEable-GPS) has been recently created. BEable-GPS enables design of gRNA for specific applications for almost every existing base editor system [163].

Importantly, designing gRNAs with a mismatched nucleotide complementary to the nucleotide mutated in the target (generated after base editing) increases efficacy of RNA editing by dCas13b-ADAR2 [142].

## 7. Conclusions and Perspectives

Many CRISPR-Cas systems have been employed by researchers to introduce various modifications into living organisms. Bioinformatics will allow discovery of numerous other systems in the coming years. Characterizing CRISPR-Cas and related systems has become the most important area of research in biology.

Genetic engineering has enabled directed modifications of CRISPR components to create optimized and highly effective gene editing approaches. Some of these tools were reviewed in this paper; many recent achievements, however, are outside the scope of this manuscript, the most notable of which includes creation of a multiplexed Cas12a-based approach for simultaneously modulating many genes to control complex biological processes with unprecedented accuracy. This multiplexing is done by a Cas12a protein that cuts a single RNA transcript into many gRNAs targeting individual targets [164]. Two groups have recently created a revolutionary approach based on dCas linked to transposases enabling highly efficient on-target integration of DNA sequences. Integrating desired DNA into the target site is a basic need of gene engineering necessary to meet many scientific and technological challenges [165,166].

New avenues in epitranscriptomics were opened by dCas tools empowered by m6A marker “writers” and m6A “erasers,” which modify the epigenetic state of RNA [167].

Visualizing target DNA and RNA sequences is another extraordinary achievement made possible by combining dCas with fluorescent proteins for in vitro and in vivo microscopy [168,169,170]. Creating cellular recorders with dCas systems like CAMERA [171] and DOMINO [172] allows writing incoming events and their parameters for detailed investigations of signaling cascades and other biological processes. CRISPR-driven evolution [132], CRISPR-diagnostics [173,174], CRISPR-biosensors [175], and many more create the foundations for the new technological era.

The utility of new CRISPR-Cas-based methods and therapeutics could be undermined by the potential off-target activity of Cas proteins, i.e., unintended binding/cutting at undesired sites. Off-target cleavage/binding may severely compromise the utility of CRISPR-Cas-based therapeutics, disrupting genes, introducing large mutations [176] and contributing to chromosome instability [177]. To reduce or even avoid potential off-target activity, a plethora of technical refinements has been made, including (a) advanced design of gRNAs using in situ CRISPR-Cas design tools (CRISPR design, E-Crisp, CROP-IT, Cas-OFFinder) [178], (b) modifications of gRNAs (truncations [179], introduction of secondary structures [180] etc.), (c) rationally engineered SpCas9 variants (eSpCas9 [181], Sp-HF1 [182], evoCas9 [183], Hypa-Cas9 [184]) with limited non-specific cleavage and off-target activity, (d) Cas proteins with altered PAM-specificity [185], (e) orthologous CRISPR-Cas systems [77,186,187], (f) engineered dCas proteins fused with FokI nuclease [188], and (g) delivery of CRISPR-Cas components as short-lived ribonucleoprotein complexes [189]. These technical achievements have minimized, but still not completely erased, potential off-target activity. Notably, off-target activity seems to be the most important issue for gene editing applications, e.g., by means of base editors (see above) or CRISPR-Cas nucleases. In contrast, off-target activity of CRISPR-Cas for epigenetic modifications does not appear to be a matter of significant concern. Off-target epigenetic modifications are usually transient and do not have an effect on transcriptional activity of off-target genes. In particular, Matharu et al. did not observe either off-target epigenetic modifications or non-target alterations in mRNA levels in mice stably transduced with AAV-CRISPRa [80]. Carefully designed gRNAs with the minimal number of predicted off-target sites and a highly specific Cas protein (rationally designed or orthologous with restrictive PAM) dramatically reduce the chances of undesired genome/epigenome modifications at non-specific loci.

To conclude, the field of CRISPR is currently evolving at a furious pace. Many years of research in biology, physics, and chemistry have been poured into the new CRISPR tools, allowing previously unfeasible biological manipulations and interventions. On the shoulders of giants, the CRISPR field is growing into one of the most powerful molecular tools. However, several major barriers stand before the CRISPR-Cas field, and the way these barriers are overcome will define the broadness and applicability of gene editing.

## Figures and Tables

**Table 1:** Epigenetic regulation by dCas-based tools. The number of (+) symbols indicates the potency of a particular dCas tool.

Target	Modification	Effect on Gene Transcription	System	Efficacy
DNA	Methylation	Supression	DNMT3A [6,7,8]	+
			DNMT3A-3L [9]	+++
			SunTag-DNMT3A [10];	+++
	Demethylation	Activation	dCas9-TET1 [8,11]	+
			dCas9-SunTag-TET1 [12]	+++
			dCas9/MS2/MCP-TET1 [13]	+++
Chromatin	Histone demethylation	Supression	dCas9-LSD1 [48]	++
	Histone acetylation	Activation	p300Core [49]	++
	Transcriptional factor recruitment	Activation	VP64 [50,52,53]	+
			VP160/VP192 [54]	++
			p65/p65-HSF1 [50,51]	+/++
			SunTag-VP64 [59]	+++
			VPR [55,56]	+++
			SunTag-p65-HSF1 [58]	++++
			SAM [51,60]	++++
			TREE [62]	++++
			Casilio [61]	+++
			Scaffold [60]	+++
		Supression	dCas9-KRAB [43]	++
			dCas9-KRAB-MeCP2 [47]	+++
			dCas9-EZH2 [34]	++
	Exogenic promoter recruitment	Activation	CMV [63]	++++

**Table 2:** Applications of CRISPRi and CRISPRa tools in different areas of research and manufacture.

**Fundamental Studies**		
Application	CRISPR tool	Target
Annotating regulatory elements	dCas9-KRAB dCas9-p300	Distal regulatory elements [69,70]
	dCas9-KRAB	Estrogen receptor enhancers [87]
Analyzing gene function	dCas9-KRAB/ dCas9-VPR	Function of *Syt1* [88], *Bdnf*, and *Reln* [89] in mammalian brain
Analyzing cell signaling	dCas9-VPR Scaffolds SAM	Generating chimeric receptors [90,91]
Identifying antiviral factors	SAM	Norovirus infection [79]
Analyzing human genome	dCas9-KRAB	CRISPRi gRNA libraries [92]
	SAM	CRISPRa gRNA libraries [92]
Annotating tumor-related factors	SAM SunTag-VP64 dCas9-KRAB	Genes involved in cancer: *Hells* [93], *Egfr* [51], *lncRNAs* [94,95,96], *Myc* [97], *Kras-*dependent genes [98]
**Creating Therapeutic Approaches**		
Application	CRISPR tool	Target
Treating infectious diseases	SAM; Scaffold (MCP-p65-HSF1)	HIV therapy by activating *BST2/tetherin* [99], *APOBEC3B* [71], and *APOBEC3G* [71]
	dCas9-p300	HBV therapy by activating *APOBEC3A, APOBEC3B, APOBEC3G, AID* [76]
	SunTag-VP64; dCas9-VPR; SAM	Reactivating HIV in a “shock-and-kill” therapeutic approach [72,73,74,75]
Treating metabolic and inflammatory diseases	dCas9-KRAB	Repressing *TNFR1*, *IL1R1*, *IL6st* [100,101,102]
	SAM	Neuro- and nephroprotection by activating *Klotho* gene [103]
	dCas9-KRAB	Repressing *Pcsk9* to reduce serum cholesterol levels [104]
	SAM dCas9-VP160	Generating insulin-producing cells by upregulating *Pdx1* or *Ins* [105,106]
Treating genetic disorders	SAM	Treating DMD by activating *Utrophin* gene [105]
	dCas9-VP64	Treating obesity by upregulating *Sim1* [80]
	dCas9-VP64	Treating Dravet syndrome by upregulating *Scn1a* [81]
	dCas9	Correcting myotonic dystrophy types 1 and 2 by blocking transcription of expanded microsatellite repeats [107]
	dCas9-VP64	Treating congenital muscular dystrophy type 1A by upregulating *Lama1* [108]
Treating cancer	dCas9-DNMT3A dCas9-KRAB dCas9-Ezh2	Repressing *Granulin* proto-oncogene [82]
	dCas9-VP64 dCas9-VPR	Activating tumor suppressors *PTEN* [83], *CHEK2* [84], *DKK3* [85]
	dCas9-VP64	Activating telomere-targeting Cas9 nuclease in cancer cells [109]
	SAM	Increased presentation of tumor antigens to immune cells [86]
Stem cell field	dCas9-VP64 SAM dCas9-p300	Generating iPSCs by inducing *KLF4*, *LIN28*, *MYC*, *OCT4*, *SOX2* [110,111,112]
	dCas9-VPR	Upregulating *NANOG* to maintain pluripotency [113]
	SAM SunTag-VP64	Differentiating stem cells into adipocytes [114], neural cells [115], pancreatic cells [116]
	SAM SunTag-p65-HSF1	Neural reprogramming by activating *Neurog2, Ascl1, Neurod1, Dkk1*, etc. [58]

**Table 3:** Applications of dCas-based editors in different areas of research and manufacture.

Aim	Deaminase Domain	Applications
Disease modeling	AID	Mutating *Bcr-Abl* gene resulting in imatinib resistance [130]
	CRISPR-X (dCas/MCP-AID)	Mutating *Psmb5* resulting in bortezomib resistance [132]
	rAPOBEC1	Mutating *Ctnnb1*, *Apc*, and *Pi3kca* genes as cancer models [147]
	TadA	Introducing SNPs to model hereditary persistence of fetal hemoglobin syndrome and hereditary haemochromatosis [134]
	rAPOBEC1 TadA	Modeling DMD and albinism by mutating *Dmd* and *Tyr* genes [148,149,150]
	Target-AID rAPOBEC1	Modeling amyloidosis by mutating *Psen1* gene [153]
	rAPOBEC1 TadA	Modeling hereditary diseases by mutating *Tia1, Lmna,* and *Dmd* genes [152]
Developing new therapies	APOBEC3A rAPOBEC1	Correcting β-thalassemia-linked mutations [154,155]
	rAPOBEC1	Correcting phenylketonuria-linked mutations [157]
	rAPOBEC1	Introducing stop codons in *Pcsk9* gene to treat atherosclerosis [158,159]
	ADAR2	Correcting mutations in *Avpr2* and *Fancc* mRNAs to treat X-linked nephrogenic diabetes and Fanconi anemia [142]
	rAPOBEC1	Treating Marfan syndrome by correcting pathogenic mutation *Fbn1^T7489C^* [156]

**Table 4:** Properties of different dCas base editing systems.

System	Change	Activity at Methylated Sites	Target	Base Editing Window
dCas9-rAPOBEC1	C→T	Weak	DNA	13–17 nt from PAM [128]
dCas9-APOBEC3A	C→T	Potent	DNA	13–18 nt from PAM [129]
dCas9-AID	C→T	Weak	DNA	16–19 nt from PAM [131]
dCas9-TadA	A→G	-	DNA	14–16 nt from PAM [134]
dCas13b-ADAR2 (RESCUE)	A→I C→U	-	RNA	-
dCas13b-ADAR2 (REPAIR)	A→I	-	RNA	-
